# Integrative indexes reveal the tolerance of winter wheat to different overwinter freezing injury

**DOI:** 10.3389/fpls.2024.1419381

**Published:** 2024-10-17

**Authors:** Lu Wu, Weidong Yu, Chen Cheng, Liping Feng, Jintao Yan, Dawei Zheng, Feiyun Yang

**Affiliations:** ^1^ China Meteorological Administration Training Center, Beijing, China; ^2^ College of Resources and Environmental Sciences, China Agricultural University, Beijing, China; ^3^ China Meteorological Administration, Henan Key Laboratory of Agro-meteorological Support and Applied Technique, Zhengzhou, China; ^4^ College of Ecology, Lishui University, Lishui, China; ^5^ Tianjin Climate Center, Tianjin, China

**Keywords:** freezing tolerance, lower temperature threshold of tiller, yield loss, soil effective negative accumulated temperature, Fv/Fm, winter wheat

## Abstract

Winter wheat (*Triticum aestivum* L.) is a crucial crop that guarantees food supply in the North China Plain (NCP). As the frequency of extreme cold events increases, it is necessary to explore the freezing resistance of different wheat varieties in order to clarify planting boundaries and help with risk assessment. In this study, 2-year controlled experiments were conducted to explore the effect of freezing temperatures (*T*_air_) and freezing durations on three winterness types. A set of indexes were used to characterize the subfreezing stress on wheat tiller, leaf, and final yield. Logistical regressions were used to quantify the temperature threshold for 10%, 30%, and 50% of freezing injury. The results showed that the lower temperature threshold of tiller (LT) varied from −9.6 to −15.9°C, −10.7 to −19.1°C and −11.4 to −21.2°C for LT_10_, LT_30_, and LT_50_, respectively. The difference between LT and yield loss (YL) indexes reduced with decreased winterness types and was −0.1 to 3.4°C, −0.7 to 2.1°C, and 0.3 to 0.9°C higher compared with YL thresholds for winterness, semi-winterness, and weak-winterness types, respectively. The average minimum soil temperature was 7.5, 4.8, and 4.2°C higher than *T*_air_ for 1-, 2-, and 3-day treatment, respectively. Soil effective negative accumulated temperature hours (TSE_h_) ranged from 6.9 to 12.0, 48.4 to 6.9, and 84.7 to 106.9°C·h for 10%, 30%, and 50% tiller mortality, respectively. Freezing treatment with *T*_air_ < −12, −9, and −8°C obviously decreased leaf Fv/Fm for the three varieties and Fv/Fm declined obviously after 5 days of recovery under field conditions. Our results provided multiple indexes for quantifying subfreezing damage in practical wheat production and could shed light on future risk assessment.

## 1 Introduction

Winter sowing crops have been found to possess a competitive advantage over spring sowing crops, primarily due to their early development in spring. This early development allows for the full utilization of spring soil moisture and leads to earlier maturation (Pirjo et al., 2011; Rizza et al., 2011). This advantage is particularly significant in the North China Plain (NCP), where the double-cropping system of winter wheat (*Triticum aestivum* L.) and summer maize is prevalent (Wang et al., 2012; Gao et al., 2019). The NCP is responsible for approximately 68% of China’s wheat production, with its planting area accounting for 58% of the nation’s total (Sheng, 2016; Hong et al., 2023). Hence, ensuring food security in this region is of utmost importance. However, the success of winter crop growing is threatened by negative climatic conditions that can result in a decrease in overwinter survival rate, crop vigor, and final yields (Licker et al., 2013; Hong et al., 2023). To counteract the potential negative effects of climate change, it has been suggested that weaker winter varieties could be planted to achieve higher yields (Liu et al., 2009; Macholdt and Honermeier, 2017), given the rising temperatures in winter and spring (You et al., 2010; Sommer and Lengfellner, 2010). However, climate change also brings about increased climatic uncertainty and a higher frequency of extreme weather events (Judy et al., 2020). For example, China has experienced extreme cold events during the past decade, in 2008, 2010, 2016, and 2017, all of which have had a significant impact on wheat cultivation. The higher spring and autumn temperatures associated with climate change can delay plant hardening and accelerate dehardening, thereby making the crop more vulnerable to cold spells for an extended period (Rapacz et al., 2014). Considering these factors, it becomes clear that the benefits of overwintering crops become more complex to assess.

Winter survival of plants in the field is a complex process influenced by multiple factors, not solely dependent on temperature (Bergjord Olsen et al., 2018; Beil et al., 2019). The ability of winter wheat to survive winter conditions is referred to as winter hardiness. Winter hardiness is a complex trait that reflects tolerance to various combinations of freezing, desiccation, anoxia, ice encasement, and resistance to disease (Armonienė et al., 2013; Zheng et al., 2018). Research has shown that winter wheat is vulnerable to low-temperature damage, from both short exposures to extreme cold and prolonged exposure to milder sub-zero temperatures (Skinner and Mackey, 2009). Furthermore, repeated cycles of freezing and thawing can exacerbate the damage (Vico et al., 2014). It is important to recognize that the degree of injury is determined by both temperature and exposure time (Skinner and Mackey, 2009; Skinner, 2014). The sensitivity to low temperatures varies among different plant organs (Gusta and Wisniewski, 2013). In order to evaluate the freezing tolerance of winter wheat, most experimental studies have focused on the LT_50_ (temperature at which 50% of the plants are killed) threshold (Armonienė et al., 2013; Fowler et al., 2014; Bergjord Olsen et al., 2018), with particular emphasis on crown tissue as it is crucial for winter survival and the regeneration of other damaged plant organs (Li et al., 2015). However, it is also important to consider yield loss (YL) as an index for evaluating the severity of cold damage (Min et al., 2020; Li et al., 2021). Freezing injury can result in significant YL, outweighing the impact of other environmental factors. To estimate freezing tolerance, chlorophyll fluorescence-based techniques have been developed as reliable, non-invasive, and easy-to-use tools (Rapacz and Woźniczka, 2009; Rapacz et al., 2015; Jaimez et al., 2023). These techniques provide researchers with indirect information about the structure and function of the photosynthetic apparatus, aiding in the assessment of freezing tolerance (Rapacz et al., 2015). It is crucial to consider the plant as a whole and its surrounding environment to gain a comprehensive understanding of the factors affecting wheat’s cold tolerance. By addressing these aspects and employing appropriate protocols, researchers can study and identify sources of improvement in cold hardiness.

Numerous investigations have been conducted to assess the cold tolerance of winter wheat varieties (Mu et al., 2015; Zheng et al., 2018). However, most of these studies have focused on the ability of the plants’ tillers/crown to withstand extremely low temperatures. Considering the changes in prewinter growing conditions due to climate change, such as the hardening process, we hypothesize that the freezing tolerance of winter wheat may have been altered. Additionally, the indexes obtained from the freezing chamber and greenhouse may not accurately reflect the field conditions, which limits their practical application (Mu et al., 2015; Zheng et al., 2018). Moreover, there have been very few studies that have evaluated the freezing tolerance of different organs of winter wheat, especially in a non-destructive manner. Therefore, the objectives of this study are twofold: (1) to assess the freezing tolerance of various organs of winter wheat using indexes that accurately correspond to field conditions, and (2) to evaluate the impact of different lengths of freezing days on the survival of winter wheat.

## 2 Material and methods

### 2.1 Experimental design

Three winter wheat varieties with different winterness types were sown in round plastic pots (diameter = 25 to 30 cm), at 2 and 1 October in 2015 and 2016 at Shangzhuang and Baodi, located in the NCP. The winterness variety “Nongda211” (ND211) is typically planted at the north region of the NCP, which can resist mild cold freezing temperatures. The semi-winterness variety “Zhengmai366” (ZM366) is usually planted at south-middle regions of the NCP. The weak-winterness variety “Yanzhan4110” (YZ4110) is normally planted at south part of the NCP, with weakest resistance to cold freezing temperatures. The under part of plastic pots was removed and then filled with perforated and well-drained sandy loam soils to allow the supply of soil water from deeper soil layers. Initially, 150 kg N/ha of compound fertilizer (N:P:K = 12:12:12) was applied. Each pot contained 25 seeds and was thinned to 20 seeds per pot after emergence, giving a plant population of approximately 280 to 400 plants/m^2^. The experiment materials were watered every 10 days to prevent from drought stress before winter. Additionally, chemical fungicides were used during the tillering stage in line with the conventional field production of winter wheat to inhibit disease or pests.

All pots were placed outdoors under field growing conditions right after sowing until the day of freezing tests so that the hardening process was similar for all the plants. The pots were placed in pits in the soil so that the plants stayed at the same level as the grain field stubble surrounding them. To prevent the pots from being firmly frozen into the surrounding soil and being impossible to remove during winter, the pits were lined with a layer of plastic mat. The low temperature always struck by late December to early February at Baodi. A layer of straw was placed on top of the field crop when minimum temperature was lower than −10°C according to the weather forecast in the 2016–2017 experiment season to prevent the crops from accumulating freezing injury due to multi-freezing events.

### 2.2 Freezing tolerance experiments

Freezing tolerance for three varieties was tested by late December and early January, when the hardening process was finished and winter wheat had gained its full freezing tolerance, with three replications for each freezing test. Wheat crown was normally located in between 2 and 3 cm following normal seed drilling (Persson et al., 2017); thus, soil temperature at 2.5 cm depth was recorded every 1 h by automatic weather stations using temperature loggers placed in the plot with an expected accuracy of ±0.1°C. The occurrence of long-lasting cold event decreased since climate warming during winter in the NCP while the short-duration low-temperature events still existed, especially in the northern part of the NCP. The frequency of cold event at our experiment site is shown in **Table 1**. Specifically, the frequency of −10°C low temperature was higher than 90% (**Table 1**). Thus, the plants were first held in the freezer at 0°C for 1 h, and temperatures were reduced at a rate of 0.8 to 2.0°C/h (targeted temperatures from −8 to −20°C for different varieties). The temperatures were kept at the target temperatures for 3 h and then gradually raised to 0°C at the same rate. After the first duration of 1 day, 1/3 of the plants were moved out. The temperature change was repeated for 2- and 3-day treatments (**Table 2**). After the freezing tests, all plants were transferred to the field and kept for regrowth until maturity. The plastic mat was removed to allow access of root to the deeper soil layer. The survival proportion (mean of three replications) was counted after 1 week of green-up stage, and plants were kept in the field until maturity. Other management practices were kept the same as the field-grown wheat, irrigated twice at the jointing stage and at the flowering stage with 60 mm each time; 75 kg N/ha of urea was spread to the surface at the jointing stage (Zadock = 31).

**Table 1:** Frequency of extreme cold weather (*T*_min_) after 1985.

	≤−20°C	≤−18°C	≤−16°C	≤−14°C	≤−12°C	≤−10°C	≤−8°C	≤−6°C
Baodi	6.1	21.2	33.3	72.7	93.9	100	100	100
ShangZhuang	0	0	9.1	36.4	63.6	90.9	100	100

**Table 2:** Experiment treatment during the 2015–2017 growing seasons.

Year	Sowing date	Variety	Freezing days	Freezing temperatures (°C)
2015–2016	2015.10.02	Nongda211	3 days	−14, −16, −18, −20
		Zhengmai366	3 days	−10, −12, −14, −16
		Yanzhan4110	3 days	−8, −10, −12, −14
2016–2017	2016.10.01	Nongda211 Zhengmai366 Yanzhan4110	1, 2, and 3 days	−10, −12, −14, −16, −18, −20
			1, 2, and 3 days	−9, −10, −11, −12, −14, −16
			1, 2, and 3 days	−8, −9, −10, −12, −14

### 2.3 Freezing tolerance indexes

To assess the damage degree of subfreezing temperatures, four different indexes were used in this paper: (i) the mortality rate of tillers (LT) was expressed as a numerical scale from 0 (no tillers were killed) to 1 (all tillers were killed) (Bergjord Olsen et al., 2018; Zheng et al., 2018) (Equation 1); (ii) the YL was the proportion of yield decreased compared to control treatment (Equation 2) (Wu et al., 2019; Dahal et al., 2021); (iii) soil degree hours as the sum of effective subfreezing temperatures at the crown depth during the freeze tests (Equations 3, 4); and (iv) the chlorophyll fluorescence index (see section 2.4) (Rapacz et al., 2015; Bai et al., 2021). The logistic function (Equation 5) was applied as it could better reproduce the effect of low temperature on crop growth (Tudela and Santibáñez, 2016; Zheng et al., 2018; Wang et al., 2021). During the 2015–2016 growing season, the three varieties experience another cold stress after the freezing test (*T*_min_ = −15.3°C for 2 days), which caused a tiller death of 0.19, 0.39, and 0.48 for the CK treatment of the three varieties, which was excluded when calculating the LT caused by the controlled freezing chamber.


(1) $$\text{LT}=\left({\text{F}}_{1}-{\text{F}}_{2}\right)/{\text{F}}_{1}$$

where F_1_ represents the tiller number before freezing experiment treatment and F_2_ is the tiller number after reviving for each treatment.


(2) $$\text{YL}=\left({\text{Y}}_{\text{ck}}–{\text{Y}}_{\text{i}}\right)/{\text{Y}}_{\text{ck}}$$

where *Y*_ck_ represents the harvested yield of control treatment (plants grown under field conditions) and *Y*_i_ is the harvested yield for each freezing treatment.


(3) $${\text{TSE}}_{\text{h}}=\sum_{i=1}^{n}\left(T_{c}-T_{i}\right)$$

(4) $${\text{TSE}}_{\text{d}}=\sum_{i=1}^{n}\left(T_{c}-T_{smini}\right)$$

where TSE_h_ is the soil effective negative accumulated temperature hours (°C•h) and TSE_d_ is the soil effective negative accumulated temperature days (°C•d). *T*_c_ is the threshold temperature of crown to withstand freezing injury (°C), *T*_i_ is the actual soil temperature at crown depth at *i*th h, *T*_smini_ is the minimum soil temperature at *i*th d. When *T*_i_ > *T*_c_ and *T*_smin_ > *T*_c_, TSE_h_ and TSE_d_ would not accumulate.


(5) $$\text{y}=\frac{1}{1+e^{aT+b}}$$

where *y* represents the dependent change (LT or YL). The temperature (–*b*/*a*) corresponded to the temperature that causes 50% death or YL.

### 2.4 Chlorophyll fluorescence index

The chlorophyll fluorescence induction parameters were determined on the second leaf of the main leaf using OS-30P (Opti-Science inc., USA) using the Fv/Fm kinetics option. Leaves were dark-adapted for 20 min with a leaf clip holder placed on the central part. By exposure to saturated red light with a photon flux density of approximately 10,000 µmol/m^2^•s to estimate the initial (F0) and maximum (Fm) fluorescence values. Fv was calculated as (Fm − F0). The parameter Fv/Fm was used to assess the efficiency of excitation energy capture by an open PSII reaction center, which is the maximum capacity of light-dependent charge separation of PSII. It was supposed to decrease after freezing injury. Firstly, Fv/Fm was measured before freezing tests, and the second measure was performed after the freezing tests when all the samples were held at 0°C for 2 h (Bai et al., 2021). It was then recorded after 1, 5, and 10 days of recovery under field conditions. Five and three replications were performed for 2015–2016 and 2016–2017, respectively.

## 3 Results

### 3.1 Evaluation of overwintering freezing tolerance of winter wheat by tiller

**Figure 1:**
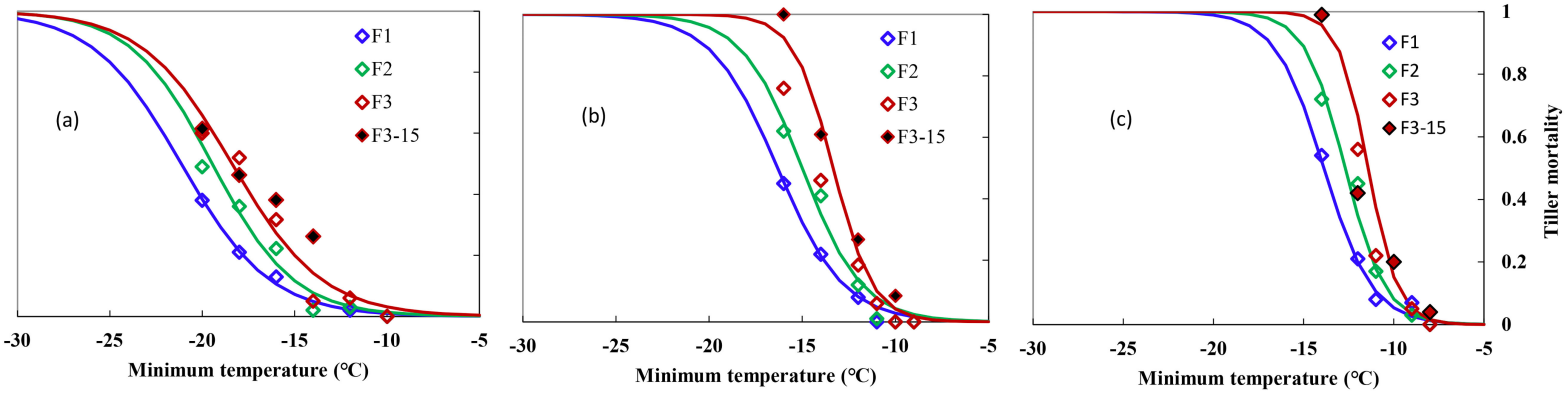
Relationship between minimum air temperature and tiller mortality during middle winter for different winterness types. **(A)** Nongda211, **(B)** Zhengmai366, **(C)** Yanzhan4110. F1 (hollow blue diamond), F2 (hollow green diamond), and F3 (hollow red diamond) indicated freezing for 1, 2, and 3 days, respectively, during the 2016–2017 growing season, F3-15 (solid black diamond) indicated freezing for 3 days during the 2015–2016 growing season.

**Figure 2:**
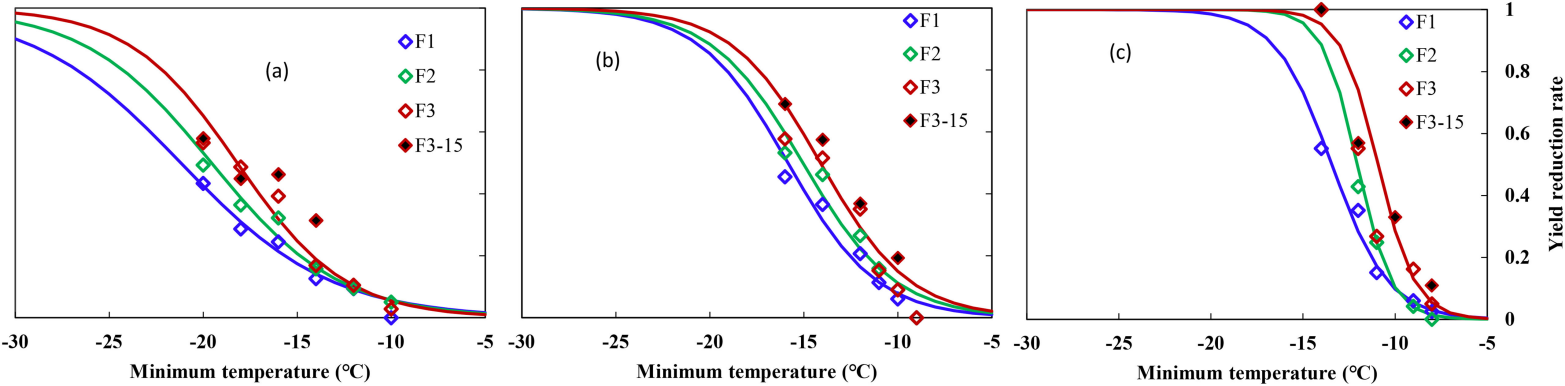
Relationship between minimum air temperature and yield loss in middle winter for winterness types. **(A)** Nongda211, **(B)** Zhengmai366, **(C)** Yanzhan4110, F1 (hollow blue diamond), F2 (hollow green diamond), and F3 (hollow red diamond) indicated freezing for 1, 2, and 3 days, respectively, during the 2016–2017 growing season, F3-15 (solid black diamond) indicated freezing for 3 days during the 2015–2016 growing season.

**Figure 1** shows the indexes of overwinter mortality, calculated in Section 2 for the 2-year experiments with different freezing temperatures and freezing days for three types of winter wheat varieties. Experiments were carried out at the end of December and at the start of January when the acclimation has completed under natural growing conditions. Winter damage increased with lower temperatures and extended freezing days. Mortality in individual trials ranged from 0 to 1 for “ZM366” and “YZ4110” during the 2-year experiments, indicating that the test conditions used were sufficient enough to result in significant different levels of survival because of variation in minimum temperature and the time held at the minimum temperature. The tiller mortality was small regardless of freezing days when *T*_min_ was higher than −14, −12, and −10°C for ND211, ZM366, and YZ4110, respectively (**Figures 1A-C**). Specifically, for “ND211”, cold stress of approximately −12°C caused 2%–6% mortality, after which tiller mortality increased from 5% to 62% with increased cold stress and prolonged freezing days (**Figure 2A**). The effect of freezing days becomes obvious when *T*_min_ ≤−16°C, where average tiller mortality increased by 12% with additional one freezing day (**Figure 2A**). Tiller mortality increased significantly with minimum temperature reduced from −12 to −16°C, while tiller mortality was ≤6% for freezing under −11°C. For cold stress lower than −14°C, average tiller mortality increased by 13.8% with an additional one freezing day for ZM366 (**Figure 2B**). It caused a tiller mortality of 3% to 100% when exposed to −9 to −14°C freezing stress for Yanzhan4110. The tiller mortality was within 25% regardless of freezing temperature or freezing days when *T*_min_ ≥−11°C while tiller mortality increased significantly when exposed to −12°C stress and reached 100% death if freezing for 3 days under −14°C treatment (**Figure 2C**).

To note, the winterness variety “Nongda211” showed greater tolerance for lower freezing damage (−13.1 to −21.2°C), followed by the semi-winterness variety “Zhengmai366” (−11.0 to −16.3°C) and the weak-winterness variety “Yanzhan4110” (−9.6 to −13.9°C). The freezing threshold temperatures of LT_10_, LT_30_, and LT_50_ ranged from −9.6 to −15.9°C, −10.7 to −19.1°C, and −11.4 to −21.2°C, respectively. As for increased freezing days, the threshold temperatures ranged from −10.9 to −21.2°C, −10.3 to −19.5°C, and −9.6 to −18.3°C, respectively. The differences between LT_30_ and LT_10_ were approximately 2.9–3.2°C, 1.4–2.4°C, and 1.4–1.8°C for the three varieties, while the differences between LT_50_ and LT_30_ were smaller, indicating that the lower the degree of low temperature, the greater the contribution of unit low temperature to mortality rate (**Table 3**).

**Table 3:** The threshold of freezing injury based on tiller mortality.

Variety	Freezing days	Equations	*R* ^2^	Freezing threshold temperatures (°C)		
				LT_10_	LT_30_	LT_50_
Nongda211	1	*y* = 1/(1 + exp(0.47 × *T*_min_ + 9.79)	0.93**	−15.9	−19.1	−21.2
	2	*y* = 1/(1 + exp(0.46 × *T*_min_ + 9.05)	0.96**	−14.7	−17.6	−19.5
	3	*y* = 1/(1 + exp(0.41 × *T*_min_ + 7.54)	0.82**	−13.1	−16.3	−18.3
Zhengmai366	1	*y* = 1/(1 + exp(0.56 × *T*_min_ + 9.15)	0.99**	−12.4	−14.8	−16.3
	2	*y* = 1/(1 + exp(0.62 × *T*_min_ + 9.31)	0.97**	−11.5	−13.6	−15.0
	3	*y* = 1/(1 + exp(0.94 × *T*_min_ + 12.56)	0.80**	−11.0	−12.4	−13.4
Yanzhan4110	1	*y* = 1/(1 + exp(0.74 × *T*_min_ + 10.31)	0.92**	−10.9	−12.7	−13.9
	2	*y* = 1/(1 + exp(0.90 × *T*_min_ + 17.18)	0.99**	−10.3	−11.8	−12.7
	3	*y* = 1/(1 + exp(1.21 × *T*_min_ + 13.85)	0.87**	−9.6	−10.7	−11.4

** indicates that the significance passes the level of 0.01, showing a very significant relationship.

### 3.2 Evaluation of overwintering freezing tolerance of winter wheat by accumulated soil effective negative accumulated temperature

**Figure 3:**
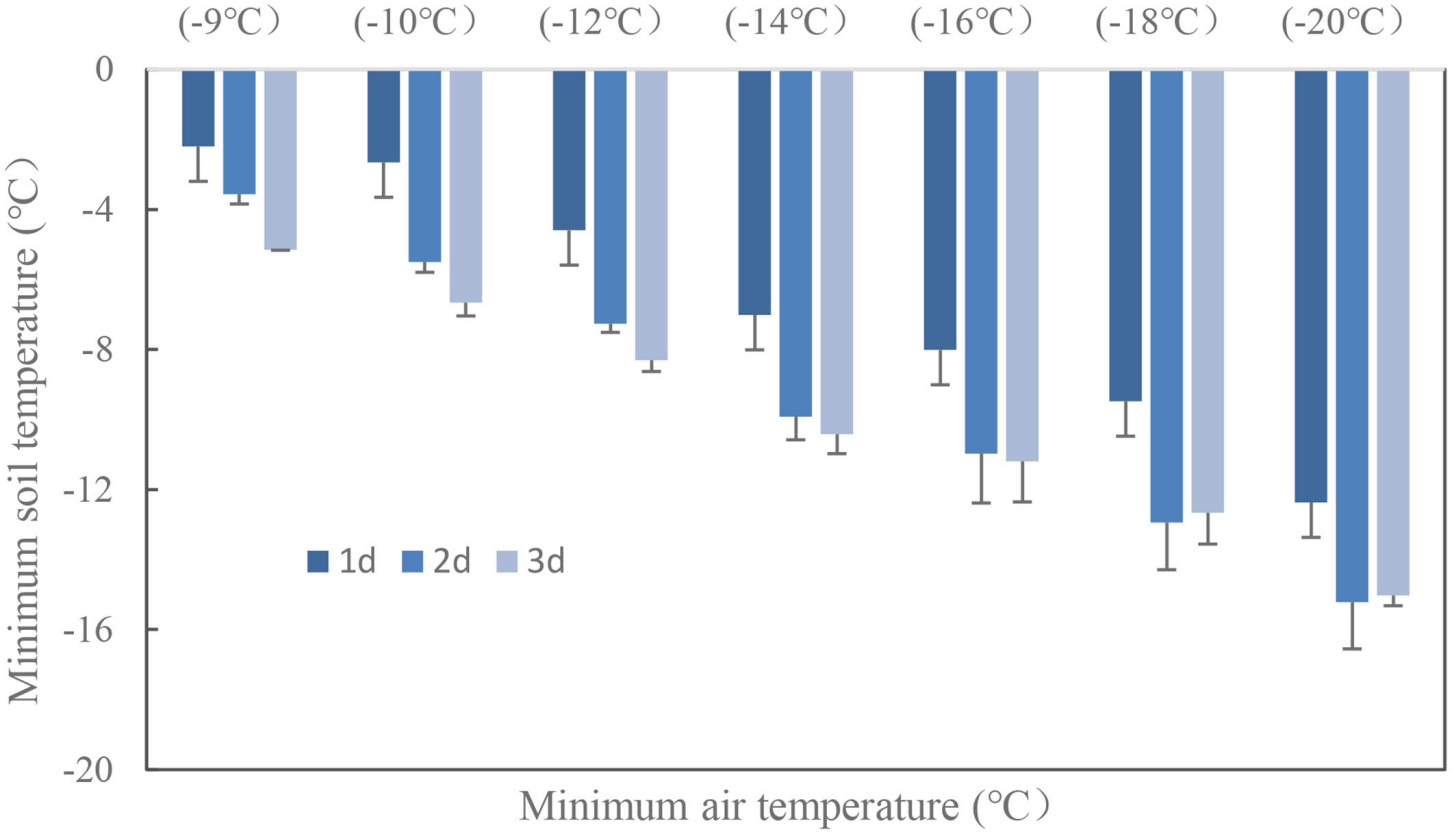
Soil minimum temperature at the crown depth with extended freezing days.

**Figure 4:**
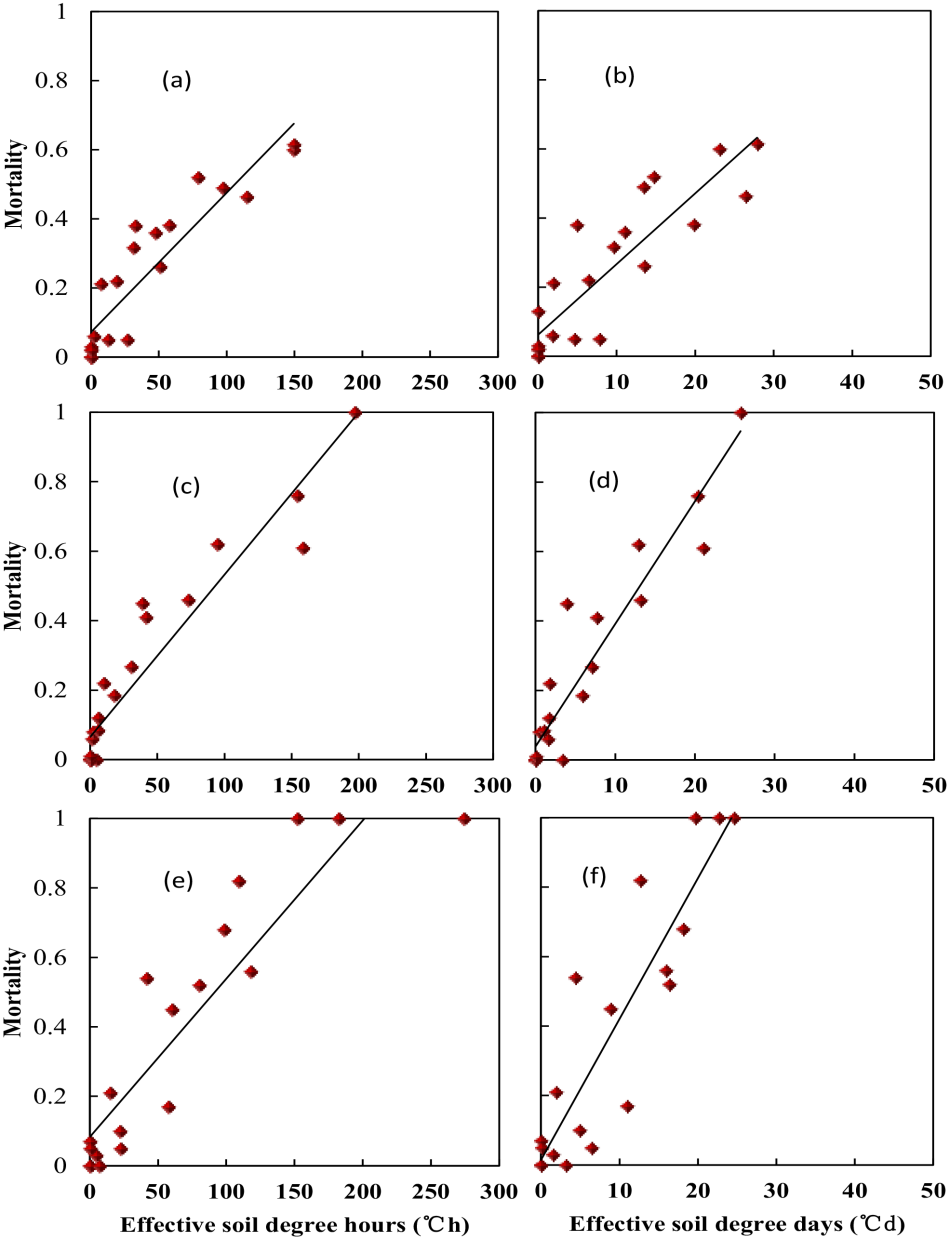
Relationship between soil effective negative accumulated temperature and tiller mortality for different variety types at middle winter. **(A)** Nongda211 (°C•h), **(B)** Nongda211 (°C•h), **(C)** Zhengmai366 (°C•h), **(D)** Zhengmai366 (°C•d), **(E)** Yanzhan4110 (°C•d), **(F)** Yanzhan4110 (°C•d).

Wheat crown tissue has the strongest tolerance for freezing injury, which was usually within 2–3 cm in the soil while temperatures fluctuated a lot in the top layer. The soil temperature at 2.5 cm was used to quantify the freezing injury on wheat plant. The average realized minimum soil temperatures were −2.2, −2.7, −4.6, −7.0, −8.0, −9.5, and −12.4°C in trials with target temperatures of −9, −10, −12, −14, −16, −18, and −20°C when freezing for 1 day. With extended freezing days, crown sensed minimum temperatures decreased, *T*_smin_ during the third freezing day would be 2.7 to 4.0°C lower than was for the first freezing day, with little difference for *T*_smin_ between the second day and the third day (**Figure 3**). The average realized minimum soil temperature was 7.5, 4.8, and 4.2°C higher than the designed minimum air temperature for the 1-, 2-, and 3-day treatment, respectively. Thus, the actual crown sensed soil temperature was used for quantifying tiller mortality instead of designed air minimum temperature irrespective of freezing days. According to experiment data, plants were killed when observed crown sensed temperature was approximately −3, −6, and −8°C for “YZ4110”, “ZM366”, and “ND211”, respectively, and thus were treated as effective freezing soil temperature for analysis later. Overwinter mortality increased linearly with increased effective negative accumulated temperature hours or days (**Figure 4**).

Specifically, soil effective negative accumulated temperature hours (TSE_h_) for “YZ4110” would be 12.0 to 84.7°C·h, which is equal to a soil effective negative accumulated temperature days (TSE_d_) of 2.0 to 11.5°C·d, resulting in 10% to 50% mortality. For “ZM366”, TSE_h_ would be 8.0 to 86.5°C·h, which is equal to a TSE_d_ of 1.8 to 12.3°C·d, resulting in 10% to 50% mortality. “Nongda211” has a stronger resistance for freezing injury with a higher value of TSE_h_ (6.9 to 106.9°C·h) and TSE_d_ (2.0 to 11.5°C·d) (**Table 4**).

**Table 4:** The threshold of freezing injury based on soil effective negative accumulated temperature hours (TSE_h_) and soil effective negative accumulated temperature days (TSE_d_).

Variety	Equations	*R* ^2^	Freezing threshold for soil effective negative accumulated temperature hours (°C•h)		
			TSE_h_ 10	TSE_h_ 30	TSE_h_ 50
Nongda211	*y* = 0.0040 × TSE_h_ + 0.0725	0.83**	6.9	56.9	106.9
Zhengmai366	*y* = 0.0051 × TSE_h_ + 0.059	0.93**	8.0	47.3	86.5
Yanzhan4110	*y* = 0.0055 × TSE_h_ + 0.034	0.86**	12.0	48.4	84.7
Variety	Equations	*R* ^2^	Freezing threshold for soil effective negative accumulated temperature days (°C•d)		
			TSE_d_ 10	TSE_d_ 30	TSE_d_ 50
Nongda211	*y* = 0.020 × TSE_d_ + 0.062	0.84**	1.9	11.9	21.9
Zhengmai366	*y* = 0.038 × TSE_d_ + 0.033	0.91**	1.8	7.0	12.3
Yanzhan4110	*y* = 0.042 × TSE_d_ + 0.015	0.78**	2.0	6.8	11.5

** indicates that the significance passes the level of 0.01, showing a very significant relationship.

### 3.3 Evaluation of overwintering freezing injury on winter wheat yield loss

Winter wheat yield was more sensitive to freezing temperatures than crown tissue, which responded negatively to decreased temperatures for all three varieties. Statistical analysis showed that minimum temperature, time held at minimum temperature, and the interaction of these two factors had a significant effect on final YL (*p* = 0.05). YL increased with prolonged freezing days, especially under lowered temperatures (**Figure 2**).

Averaged YL was <10% when *T*_min_ were higher than −12°C, 10%–20% when suffering from cold stress of −14 to −16°C, while a freezing stress of −20°C resulted in over 50% of YL for ND211. Specifically, a cold stress of approximately −12°C caused 10% YL, after which YL increased from 12.6% to 57.9% with increased cold stress and prolonged freezing days (**Figure 2A**). The effect of freezing days becomes obvious when *T*_min_ ≤14°C, where average YL increased by 8% with one additional freezing day (**Figure 2A**). YL increased significantly with minimum temperature reduced from −11 to −16°C, while YL was approximately 10% for freezing under −10°C for ZM366. For cold stress lower than −12°C, average YL increased by 7% with one additional freezing day (**Figure 2B**). It caused a YL of 4% to 100% when exposed to −9 to −14°C freezing stress for Yanzhan4110. The YL was within 60% regardless of freezing temperature or freezing days while YL increased significantly when exposed to −14°C stress and reached 100% death if freezing for 2 and 3 days (**Figure 2C**).

Specifically, the freezing threshold temperatures of YL_10_, YL_30_, and YL_50_ ranged from −11.9 to −21.3°C, −8.9 to −15.9°C, and −8.7 to −13.4°C for ND211, ZM366, and YZ4110, respectively. As for increased freezing days, the threshold temperatures ranged from −8.7 to −12.5°C, −10.1 to −17.9°C, and −10.9 to −21.3°C for freezing 1, 2, and 3 days, respectively. The differences between YL_30_ and YL_10_ were approximately 3.8–5.4°C (ND211), 3.1–3.2°C (ZM366), and 1.3–2.0°C (YZ4110) for the three varieties, while the differences between YL_50_ and YL_30_ were smaller (2.5–3.4°C, 2.0–2.1°C, and 0.8–1.3°C), indicating that the lower the degree of low temperature, the greater contribution of unit low temperature to YL (**Table 5**).

**Table 5:** The threshold of freezing injury based on yield reduction.

Variety	Freezing days	Equations	*R* ^2^	Freezing threshold temperatures (°C)		
				YL_10_	YL_30_	YL_50_
Nongda211	1	*y* = 1/(1 + exp(0.251 × *T*_min_ + 5.316)	0.98**	−12.5	−17.9	−21.3
	2	*y* = 1/(1 + exp(0.294 × *T*_min_ + 5.774)	0.98**	−12.1	−16.7	−19.6
	3	*y* = 1/(1 + exp(0.377 × *T*_min_ + 6.903)	0.95**	−11.9	−15.7	−18.2
Zhengmai366	1	*y* = 1/(1 + exp(0.423 × *T*_min_ + 6.702)	0.94**	−10.7	−13.8	−15.9
	2	*y* = 1/(1 + exp(0.409 × *T*_min_ + 6.158)	0.94**	−9.8	−13.0	−15.1
	3	*y* = 1/(1 + exp(0.422 × *T*_min_ + 5.951)	0.87**	−8.9	−12.1	−14.1
Yanzhan4110	1	*y* = 1/(1 + exp(0.647 × *T*_min_ + 8.689)	0.98**	−10.1	−12.1	−13.4
	2	*y* = 1/(1 + exp(1.056 × *T*_min_ + 12.730)	0.99**	−10.0	−11.3	−12.1
	3	*y* = 1/(1 + exp(0.811 × *T*_min_ + 9.129)	0.88**	−8.7	−10.1	−10.9

** indicates that the significance passes the level of 0.01, showing a very significant relationship.

### 3.4 Evaluation of overwintering freezing injury on winter wheat by Fv/Fm

**Figure 5:**
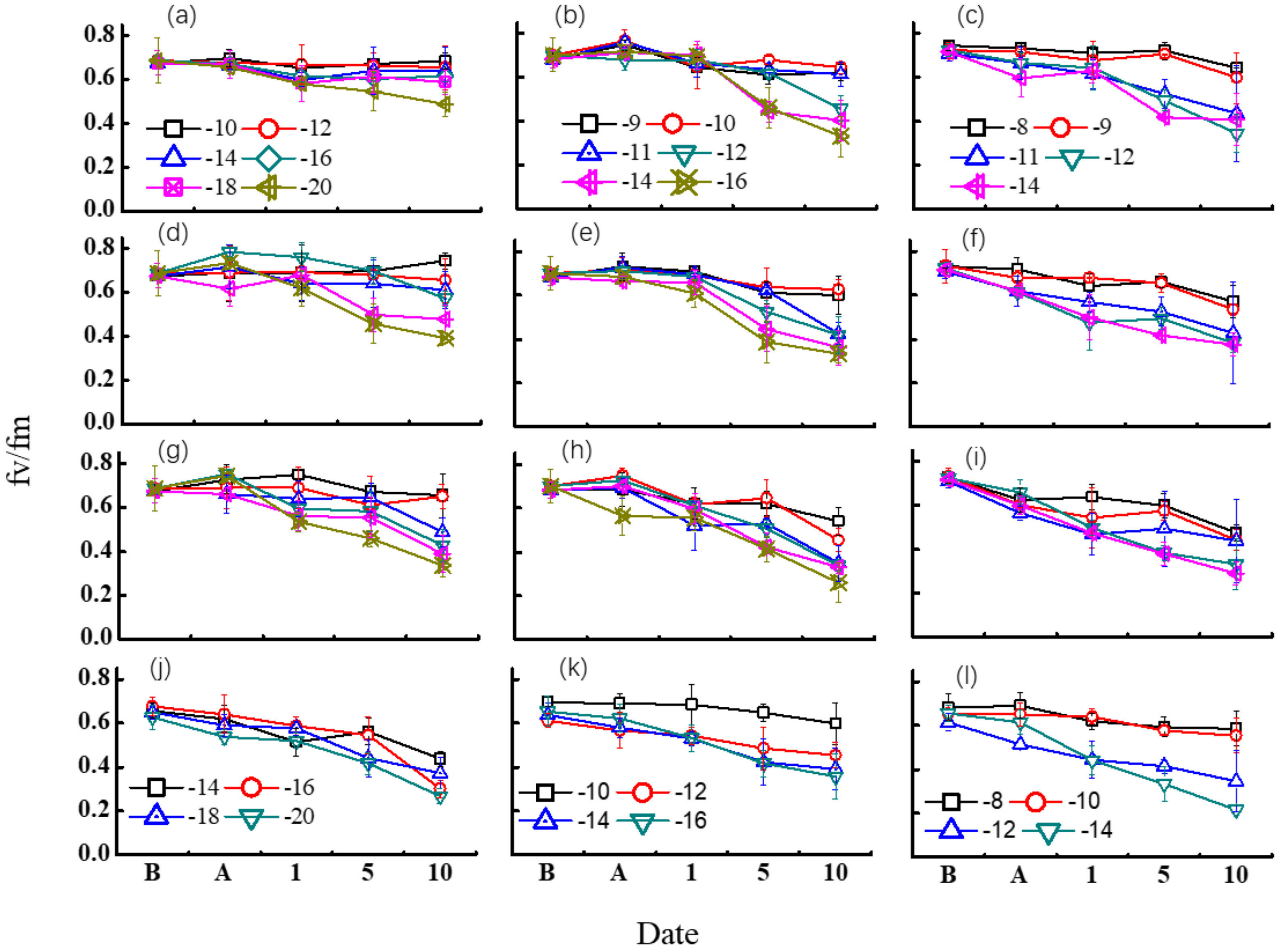
Effect of different freezing treatments on Fv/Fm ratio for different variety types in middle winter. The first three rows indicated the 2015–2016 growing season. **(A–C)** indicated freezing for 3 days in 2015 for “Nongda211”, “Zhengmai366”, and “Yanzhan4110”, respectively. **(D–F)** indicated freezing for 1 day for “Nongda211”, “Zhengmai366”, and “Yanzhan4110” during the 2016–2017 growing season, respectively. **(G–I)** indicated freezing for 2 days for “Nongda211”, “Zhengmai366”, and “Yanzhan4110” during the 2016–2017 growing season, respectively. **(J–L)** indicated freezing for 3 days for “Nongda211”, “Zhengmai366”, and “Yanzhan4110” during the 2016–2017 growing season, respectively. B means before treatment; A means after treatment; 1, 5, and 10 mean recovery after 1, 5, and 10 days, respectively.

**Figure 5** shows the changes of Fv/Fm under diverse freezing treatments. Winterness variety exhibited evident advantage over the other two varieties. For most of the treatments, no obvious trend was found after freezing tests, but it caused a dramatic decrease of Fv/Fm after 5 days of recovery in the field for most of the cases, while the Fv/Fm response was different among different freezing days.

For “ND211”, cold stress of 1 day did not have a significant influence on Fv/Fm except for −20°C. The Fv/Fm value fluctuated for other temperature treatments, while it decreased after 5 days of recovery when freezing at −20°C. Although the Fv/Fm value fluctuated after 1 day of recovery for the treatment of freezing for 2 days, a significant drop was detected for −18 and −20°C treatments after 5 days of recovery, and for −16°C after 10 days of recovery. Freezing for 3 days with a temperature below −14°C resulted in a major fall of Fv/Fm after 1 day of recovery. It seemed that the effect of low temperature was reversible when freezing at −10°C regardless of freezing days (**Figures 5A, D, G, J**).

The Fv/Fm values showed a slight increase after freezing treatment except freezing at −16°C for 3 days for “ZM366” for the 2016–2017 growing season. No obvious difference was observed for each low-temperature treatment after 1 day of recovery for freezing by 1 or 2 days, but after 5 days of recovery, the difference was apparent at −14°C and −12°C for 1-day and 2-day treatments, respectively. The Fv/Fm value was half of that before freezing after 10 days of recovery. When suffering at a temperature higher than −10°C for 3 days, Fv/Fm did not decrease much after 5 days of recovery for both experimental years. The injury was obvious after 10 days of recovery, with Fv/Fm valued from 0.38 to 0.45. It revealed that the effect of low temperature was irreversible with low-temperature treatment from −10 to −16°C regardless of freezing days (**Figures 5B, E, H, K**).

The Fv/Fm did not change much after freezing test both for 1-day and 2-day treatment for “Yanzhan4110”. The lowering of Fv/Fm was already evident after 5 days of recovery for temperature <−10°C. The injury was distinct after freezing for 3 days for both growing seasons, which continued to decrease under field recovery. The harm of cold stress was clear when *T*_min_ < −12°C (**Figures 5C, F, I, L**).

## 4 Discussion

In this study, a set of freezing indexes was applied to evaluate the sub-freezing injury on three types of field-grown winter wheat in the NCP. Freezing tolerance was determined by LT, accumulated soil effective negative accumulated temperature hours/days (TSE_h_ and TSE_d_), YL, and chlorophyll fluorescence Fv/Fm of plant samples, which were cold acclimated under natural conditions. As concerns had been stated that current experiments did not reflect true complexity of cold acclimation (Gusta and Wisniewski, 2013), plants cold acclimated under natural conditions seem to be a better way to reflect the plants suffering under field conditions (Rapacz et al., 2015). Previous studies showed that cold tolerance gained under controlled environment was stronger than that gained under field conditions (Rapacz et al., 2015; Short and Wolyn, 2022), which limit the prevailing of these indexes in reality. Actually, existing studies always put the after-treatment plants in the greenhouse, where the circumstantial environment was totally different from the field. Thus, in our experiment, we put the plants in the field after freezing treatment (with no additional freezing threat) to obtain disaster indicators that are more in line with practical production applications. Additionally, the existing studies mainly emphasize the temperature indicator while we tried to develop an index system that focuses on varied organs and quantifying methods.

### 4.1 The employment of multi-index thresholds for quantifying freezing injury

When evaluating the potential risks of winter injury, the LT_50_ parameter has commonly been utilized to quantify the likelihood of damage occurring when temperatures reach detrimental levels. This approach offers a straightforward and easily understandable method for characterizing the frequency at which harm may be expected to manifest. However, it has been argued that the 50% threshold for injury may be excessive for agronomic planting areas (Waalen et al., 2011; Vico et al., 2014). Furthermore, LT_50_ does not account for the severity of the damage, as it solely indicates whether or not damage has occurred. Consequently, this approach may yield similar risk levels across different locations where the damaging temperature has been reached (Bergjord Olsen et al., 2018). To address these limitations, a novel assessment method has been proposed in this study. The S curve was employed to assign slight injury, moderate injury, and severe injury ratings of 10%, 30%, and 50%, respectively, to evaluate a specific subfreezing damage for each cultivar. Accordingly, it has been suggested that considering 5% of kill as slight injury would align with the established standards for agrometeorological disasters in China. However, it should be noted that both overestimation and underestimation may occur at the extremities of the response curve (Tudela and Santibáñez, 2016).

Actually, the LT_50_ promoted by this study was a lot higher compared with the existing studies (Mu et al., 2015; Zheng et al., 2018). In our study, all wheat varieties underwent cold resistance training under natural conditions. After the low-temperature treatment, the wheat plants were placed in the field to reach their final maturity. However, wheat growth in the field is a complex process that involves various factors, including exposure to low temperatures (Gusta and Wisniewski, 2013). When wheat seedlings are exposed to freezing damage and left in the field, external factors such as strong winds and sunny days can exacerbate dehydration and lead to plant death. As a result, the freezing resistance index obtained from field experiments generally appeared weaker compared to those from greenhouse experiments (Mu et al., 2015; Zheng et al., 2018). For instance, Mu et al. (2015) reported that the LT_50_ values for winter wheat varieties were −21.1°C, −18.5°C, and −14.7°C for soil temperature, whereas we reported the similar threshold range using air temperature but not soil temperature. The reason may be that the freezing resistance of tillers is likely to be weaker compared to that of crown nodes/whole plants. Furthermore, climate change has significantly impacted China’s weather patterns since 1980, resulting in the absence of stable snow cover in the NCP during winter (Zhang et al., 2015). Many freezing damage indexes in China are based on research conducted before the 1980s when winter wheat varieties exhibited stronger resistance (Zheng, 1981; Gong et al., 1982). Moreover, in field production, the cumulative effects of repeated mild low-temperature threats throughout the long winter, along with temperature fluctuations and nutrient consumption, gradually weaken and relieve resistance over the entire overwintering period (Ferrante et al., 2024). Dry soil surfaces in winter, combined with strong winds and soil cracking, can lead to water loss and the natural death of overwintering seedlings. Therefore, even if the temperature in the field is slightly higher than the critical lethal temperature identified in the greenhouse, severe freezing damage and the death of seedlings can still occur.

### 4.2 The promotion of crown perceived soil temperature as freezing index

The soil temperature was less utilized when quantifying crop growth, since freezing tolerance is often revealed using LT_50_ of minimum air temperature in most winter damage studies. However, recent research has highlighted the significant impact of the duration of exposure to the minimum temperature on freezing tolerance (Skinner and Mackey, 2009; Skinner and Bellinger, 2011; Skinner, 2014). Furthermore, variations in soil temperature under different crop growing conditions have limited the applicability of air temperature measurements in diverse production areas (Yang et al., 2021). To address these limitations, researchers have explored the use of the sum of soil temperature below a threshold (often 0°C) to assess prolonged exposure to relatively milder temperatures. Nonetheless, the absence of cultivar-specific parameters has hindered the practical application of this approach (Vico et al., 2014). In this study, we propose the use of TSE, which incorporates cultivar-specific threshold temperatures derived from experimental results. This index provides a more accurate and comprehensive assessment of freezing tolerance. The TSE indexes are recommended for locations where shallow soil temperatures can be obtained, as the translation of air temperature to soil temperature can vary under different weather conditions (e.g., sunny, cloudy, rainy, or with snow cover). Considering the significant geophysical differences in winter wheat cultivation in the NCP, the varying seeding dates pose an additional concern. The density of plant cover before winter can greatly affect soil temperature dynamics, with denser covers resulting in less drastic changes compared to plants sown later with fewer leaves and a colder crown sensed temperature (Luo et al., 2018; Islam et al., 2022). The freezing of soil is a rapid process, and the minimum soil temperature realized became colder with prolonged freezing time (**Figure 3**). Occasionally, abnormal points may be observed in **Figure 4** for all cultivars. It is worth noting that smaller accumulated TSE values resulted in higher death rates, while some larger TSE values led to lower death rates. This phenomenon can be explained by the freezing tolerance mechanism in wheat, which is activated only after exposure to a sufficiently low temperature for a sufficient duration (Skinner, 2014). Assessing the risks based on TSE provides a more stable comparison of freezing conditions across diverse climatic conditions. Therefore, incorporating the TSE indexes into winter damage studies can enhance our understanding of freezing tolerance in crops and inform decision-making regarding cultivation practices.

### 4.3 The application of yield loss index to measure low-temperature damage

Currently, the focus of most indexes lies in tiller death, disregarding the growth of after-treatment. However, in reality, YL plays a significant role in ensuring food security. The index of YL exhibits a similar trend to LT (low temperature), although the threshold temperature differs a lot. The reason may that invisible injury may occur in the growth cone, resulting in adverse effects on green-up growth. This effect is particularly severe in later-seeded plants in actual production, as excessive biomass consumption due to freezing injury leaves insufficient biomass for re-tillering, even if the crown has not been completely freezing to death. Shoot regrowth reflects both the number of surviving plants and their vigor (Waalen et al., 2011; Kinugasa and Gantsetseg, 2023); thus, excessive consumption during winter can have a considerable impact on later yields. Existing studies pointed out that freezing damage was composed of two components: the proportion of plants destroyed by freezing and the YL observed in damaged but surviving plants (Veisz et al., 2001). The side spikes, rather than the main spike, tend to be more susceptible to freezing, which cannot be accurately reflected solely by LT_50_. Additionally, the root systems of cereals are more vulnerable to freezing than the shoots, and cold freezing temperatures between −5 and −9°C can cause their death. The lack of root initiation in the field rather than just the death of a proportion of crowns are the cause of winter kill, and thus, differences existed between LT and YL.

### 4.4 The utilization of the non-destructive index for assessing low-temperature threats

The complexity of the response to cold damage is often underestimated (Gusta and Wisniewski, 2013), highlighting the need for considering the effect of cold damage on the entire plant. To evaluate the cold tolerance of leaves, this study employed the chlorophyll fluorescence Fv/Fm as a reliable, non-invasive, and easy-to-use tool (Rapacz et al., 2015; Jaimez et al., 2023). Our results revealed no distinct difference after the freezing tests, but a noticeable change in Fv/Fm was observed after a 5-day recovery period. The selected value of 5 days of recovery as a freezing indicator was supported by existing studies (Rapacz, 2007; Rapacz and Woźniczka, 2009), which stated some concerns on using Fv/Fm as a wheat freezing indicator when measures were made directly after freezing. Additionally, Rapacz et al. (2015) found that the highest correlations were obtained when chlorophyll fluorescence measurements were taken in mid-winter, which coincides with the time frame of our experiment. Although this indicator represents photo-inhibitory damage with a relatively short time lag, it provides farmers with the advantage of early detection of freezing injury before the next spring, enabling them to implement remedial measures to mitigate possible significant damage (Rizza et al., 2011; Wang et al., 2016) and adoption of agronomic management practices to promote re-tiller growth and compensate for YL.

The freezing stress experiments described in this paper were conducted in a controlled freezing chamber without snow cover on the surface. In field conditions, however, snow cover can sometimes be present, although it is not stable in the NCP, particularly under the influence of climate change. In the NCP, snow cover is typically shallow and does not significantly contribute to soil warming when the snow depth is less than 5 cm (Persson et al., 2017). Under the impact of climate change, the occurrence of freeze–thaw overwinter damage is on the rise (Bergjord Olsen et al., 2018); we confronted this kind of injury in the 2015–2016 experiment, and the mechanism of this type of damage should be further studied in the future. Moreover, it would be ideal to treat all three varieties at the same temperature, even though this would have increased the number of treatment combinations in the study; thus, we suggest future studies to pay attention to this kind of shortcoming.

## 5 Conclusion

This study aimed to investigate the tolerance thresholds of various winter wheat organs to freezing injury using a controlled freezing chamber. Following exposure to sub-freezing temperatures, the treated plants were grown in the field until maturity. The relationship between air temperature, soil temperature, and plant damage (10%, 30%, and 50%) was established, and the effects of temperature threats on leaf fluorescence (Fv/Fm) were quantified. The freezing tolerance varied among different plant organs. The lower-temperature threshold of tiller (LT) ranged from −9.6 to −15.9°C, −10.7 to −19.1°C, and −11.4 to −21.2°C for LT_10_, LT_30_, and LT_50_, respectively. The difference between LT and YL indexes decreased with less winterness, with values of −0.1 to 3.4°C, −0.7 to 2.1°C, and 0.3 to 0.9°C higher for winterness, semi-winterness, and weak-winterness types, respectively. The minimum soil temperature decreased with additional freezing days under the same target air temperature. The hourly soil effective negative accumulated temperature (TSE_h_) ranged from 12.0 to 84.7°C·h (YZ4110), 8.0 to 86.5°C·h (ZM366), and 6.9 to 106.9°C·h (ND211) for 10% to 50% tiller mortality. In most treatments, no significant trend was observed in Fv/Fm following freezing tests, but a dramatic decrease was observed after 5 days of recovery in the field. Overall, this study provides easily recorded indexes for assessing freezing injuries in winter wheat organs and demonstrates the non-destructive use of Fv/Fm in advance for freezing quantification and enable them to implement remedial measures to mitigate the possible significant damage.

## Data Availability

The data presented in this study are available on request from the corresponding author. The data are not publicly available because they need to be used in future work.
